# Evaluation of Factors Associated with Intracranial Hemorrhage in Patients Presenting to the Emergency Department with Head Trauma: A Prospective Observational Study

**DOI:** 10.3390/jcm14248735

**Published:** 2025-12-10

**Authors:** Kubra Parpucu Bagceci, Pakize Gozde Gok, Filiz Baloglu Kaya, Nurdan Acar, Engin Ozakin, Evvah Karakilic, Mustafa Emin Canakci

**Affiliations:** 1 Department of Emergency Medicine, Yunus Emre State Hospital, 26190 Eskisehir, Türkiye; 2 Department of Emergency Medicine, Faculty of Medicine, Eskisehir Osmangazi University, 26040 Eskisehir, Türkiyenurdanergun@gmail.com (N.A.);

**Keywords:** emergency, head trauma, intracranial hemorrhage, novel oral anticoagulants

## Abstract

**Background:** Head trauma is one of the most common causes of morbidity and mortality in emergency departments. It is essential to understand the factors leading to intracranial hemorrhage (IH) in order to improve outcomes and reduce healthcare costs. The aim of this study was to evaluate the factors associated with IH in patients presenting to the emergency department with head trauma. **Methods:** This prospective observational study was conducted between 27 February 2019 and 27 February 2020 at the emergency department of Eskisehir Osmangazi University. Patients aged ≥18 years with head trauma were included. Data were collected from medical records, with follow-up information obtained via telephone or hospital revisits. Analyses were performed using the chi-squared test in IBM SPSS Statistics (version 21), with *p* < 0.05 being considered significant. **Results:** Of the 556 patients, 59.7% were male, and 40.3% were female, with an average age of 45.9 years (SD 21.5). Intracranial hemorrhage (IH) was more prevalent in patients aged over 60 years, with comorbidities, taking medication (especially anticoagulants and non-vitamin K antagonist oral anticoagulants [NOACs]), with altered consciousness, pathological neurological findings or experiencing non-minor trauma. Prolonged activated partial thromboplastin time and new or increased hemorrhage on follow-up CT scans were associated with IH. **Conclusions:** Advanced age, comorbidities, and anticoagulant use were key factors associated with intracranial hemorrhage (IH) in patients with head trauma. Further studies should assess predictors of mortality and outcomes.

## 1. Introduction

Head trauma is a general term that includes trauma to the skull, brain, scalp, and soft tissue in the facial region [1]. It has been reported that there are an average of 1.1 million emergency department (ED) visits annually due to head trauma, with 21% of these cases requiring hospitalization and 4% resulting in mortality [2]. Most patients with severe head trauma and more than half of those with moderate head trauma experience permanent disability or loss of baseline functional capacity [3].

The most significant consequence of head trauma caused by blunt or penetrating mechanisms is traumatic brain injury [1]. This condition accounts for approximately 60% of trauma-related deaths and nearly 90% of pre-hospital deaths [4,5].

Intracranial hemorrhage (IH), which is a significant outcome of traumatic brain injury, is also a severe complication of antithrombotic medications [6]. Studies have reported an increased risk of acute and delayed IH after head trauma in patients using anticoagulants [4,7]. Today, the rise in diseases requiring antiplatelet/anticoagulant medications, particularly among elderly patients, combined with their higher potential for trauma, increases the risk of hemorrhage as well as morbidity and mortality [7,8].

It is known that a significant portion of mortality in head trauma patients occurs within the first 24 h, making the identification of primary injuries and prevention of secondary injuries in the ED the primary goal [9].

This study aimed to examine cases presenting to the ED due to head trauma and to evaluate the factors influencing the presence of IH in this patient group.

## 2. Materials and Methods

### 2.1. Study Design and Population

This single-center, prospective, observational study was conducted between 27 February 2019 and 27 February 2020, at the ED of Eskisehir Osmangazi University The study included patients aged ≥ 18 years who presented to the ED due to head trauma and provided written informed consent for participation, either themselves or through a first-degree relative. Patients < 18 years of age and pregnant individuals were excluded from the study.

### 2.2. Data Collection

The demographic characteristics of the cases, their complaints at the time of ED admission, trauma mechanisms, comorbidities, medication use, clinical findings at admission, and Glasgow Coma Scale (GCS) scores were obtained from the hospital information system records. In addition, coagulation parameters, blood test results that could influence consciousness (e.g., glucose, ethanol), and the findings of imaging studies were evaluated. Factors affecting the neurological outcomes of the cases, as well as their neurological survival at the 6th hour, 24th hour, and 30th day following ED admission, were assessed. For neurological survival evaluation, GCS was used for the 6th hour and 24th hour, and the Glasgow Outcome Coma Scale was used for the 30th day. For the 30th-day evaluations, cases were contacted by phone, or if they had returned to the hospital, their records were reviewed.

### 2.3. Measurements

For the assessment of vital signs, normal values were defined as systolic blood pressure (SBP) 90–120 mmHg, diastolic blood pressure (DBP) 60–80 mmHg, pulse 60–100 beats/min, respiratory rate 12–20 breaths/min, body temperature 36.0–37.2 °C, and oxygen saturation (SPO_2_) ≥ 95% [10,11,12]. Cases with a GCS score of 13–15 were classified as minor head trauma, while those with a score ≤ 12 were classified as non-minor head trauma [13]. Laboratory results of the cases were evaluated using threshold values defined for secondary injuries in head trauma (glucose 80–180 mmol/L, ethanol ≥ 50 mg/dL, international normalized ratio (INR) < 1.4, activated partial thromboplastin time (aPTT) 22–34 s, hemoglobin (Hb) > 7 g/dL, hematocrit 36–47%, and platelets > 75 × 10^3^/μL) [8].

### 2.4. Statistical Analysis

Data analysis was performed using IBM SPSS statistical software (version 21). Descriptive statistics were presented as mean, standard deviation, median, minimum, and maximum for continuous variables and as frequency and percentage for categorical variables. The Chi-square test was used to compare categorical variables. The statistical significance level was accepted as *p* < 0.05.

### 2.5. Ethical Approval

Ethical approval for the study was obtained from the Eskişehir Osmangazi University Clinical Research Ethics Committee (14 February 2019/31).

## 3. Results

### 3.1. Descriptive Features

During the study period, 1453 cases were presented to the ED due to head trauma. Of these, 895 cases were excluded due to being <18 years old, and 2 cases were excluded due to pregnancy. Among the 556 cases included in the study, 59.7% (*n* = 332) were male. The mean age of the cases was 45.9 ± 21.5 years (range: 18–94), with a median age of 42 (Q1–Q3: 26.0–64.8). The most common reason for ED admission was falls, accounting for 34.0% (*n* = 189) of cases. A history of comorbidities was present in 34.9% (*n* = 194) of cases, with hypertension being the most common comorbidity (*n* = 113). Among the cases, 180 individuals (34.2%) were chronic medication users. The medications used were antihypertensives (31.2%, *n* = 110), antithrombotics (30.7%, *n* = 109), antidiabetics (9.1%, *n* = 32), antiarrhythmics (8.2%, *n* = 29), and other medications not affecting outcomes in head trauma cases (16.7%, *n* = 59). Of the 109 patients using antithrombotic agents, 82 were on antiplatelets (69 on aspirin [ASA], 25 on P2Y12 inhibitors, and 12 on both ASA and P2Y12 inhibitors), 29 were on anticoagulants (19 on warfarin, 8 on novel oral anticoagulants [NOACs], and 2 on low molecular weight heparin), and 2 were on both antiplatelets and anticoagulants (one on ASA and warfarin and one on a P2Y12 inhibitor and NOAC).

The median SBP at admission was 120.0 mmHg (min: 70.0, max: 200.0), and the median DBP was 71.0 mmHg (min: 50.0, max: 100.0). The median pulse rate was 84.0 beats/min (min: 60.0, max: 125.0), the median respiratory rate was 20.0 breaths/min (min: 14.0, max: 24.0), the median body temperature was 36.0 °C (min: 36.0 °C, max: 37.2 °C), and the median SpO_2_ was 98.0% (min: 70.0, max: 99.0).

At the end of ED follow-up, it was determined that 29 cases (5.2%) were admitted to the ward, 19 cases (3.4%) were admitted to the intensive care unit, and 3 cases (0.5%) died in the ED.

Survival evaluation of the cases showed that 3 deaths occurred within 6 h, an additional 1 death within 24 h, and another 1 death by the 30th day.

### 3.2. Presence of Hemorrhage on Brain Computed Tomography (CT) at Admission and Associated Factors

IH was detected in 18 (4.5%) of the 398 cases that underwent brain CT. A statistically significant association was found between the presence of hemorrhage and being ≥60 years old, having comorbidities, using any medication, using anticoagulant drugs, using NOAC, experiencing a traffic accident, and the presence of non-minor head trauma (Table 1).

No statistical differences were found in vital signs (SBP, DBP, pulse, respiratory rate, SpO_2_) or emergency laboratory values (glucose, ethanol, INR, aPTT, hemoglobin, and platelets) at the time of admission with respect to the presence of hemorrhage on brain CT (*p* > 0.05 for each parameter). A statistically significant association was observed between the presence of IH on brain CT at admission and altered consciousness at presentation, pathological findings on central nervous system examination, and changes observed during physical examinations at the 6th hour, 24th hour, and 30th day (Table 2).

Patients with hemorrhage on admission brain CT were more likely to require hospitalization and had higher rates of mortality (Table 3).

### 3.3. Increased Hemorrhage/Presence of New Hemorrhage on Follow-Up Brain CT and Contributing Factors

Of the 398 patients who underwent brain CT at ED admission, follow-up brain CT was performed in 41 patients (10.3%). Follow-up brain CT scans were performed at 6, 12, and 24 h after the initial ED admission. Reasons for follow-up brain CT were the presence of new symptoms (26 patients) and initiation of new anticoagulant therapy (15 patients). Among the patients with IH on admission brain CT who also underwent follow-up CT, increased hemorrhage was observed in 4 cases. New IH was detected in 2 cases.

No significant difference was found in antithrombotic drug use between those with new/increased hemorrhage and those without (*p* = 0.421). When the relationship between laboratory values at the time of ED admission and the presence of increased/new hemorrhage on follow-up brain CT was examined, aPTT > 34 s was found to be significantly associated (Table 4).

## 4. Discussion

Head traumas are frequently encountered in EDs and cause significant mortality and morbidity. Therefore, it is essential to identify factors that negatively affect the prognosis of head trauma patients and lead to IH. Early and effective intervention in this patient group is critical for improving survival rates and reducing healthcare costs.

There are studies in the literature reporting that head traumas and IH are more common in males [14,15]. However, in the present study, no gender-based difference in IH was observed among head trauma patients. This finding may be attributed to the limited number of head trauma patients included in the analysis.

Advanced age is considered a risk factor for IH [14]. Numerous studies have also highlighted the adverse impact of advanced age on the prognosis of head trauma patients [16,17,18,19,20]. A meta-analysis by Hashmi et al. reported higher rates of hemorrhage and mortality in elderly patients following head trauma [19]. Similarly, Mosenthal et al. found that subdural hematomas were more prevalent in older patients with traumatic brain injury [20]. Conversely, Sadegh et al. reported no age-related differences between head trauma patients with abnormal and normal CT findings [21]. In the present study, IH was more frequently observed in patients aged ≥ 60 years. The increased incidence of IH in this age group may be due to the greater frailty of elderly individuals and the more severe outcomes of trauma in these patients.

Certain chronic conditions, such as hypertension, are significant risk factors for spontaneous IH [14]. Additionally, chronic illnesses negatively affect survival and recovery in head trauma patients, worsening the prognosis of IH [9]. Consistent with the literature, the present study found that IH was more common in patients with comorbid conditions. This result may be attributed to impaired physiological responses to trauma and the use of medications that increase bleeding susceptibility in patients with chronic diseases.

Chronic medication use, particularly antithrombotic drugs that interfere with the coagulation cascade, is a factor that exacerbates bleeding in head trauma patients [22]. The risk of bleeding, the most serious complication of antithrombotic drugs, is further heightened after any head trauma [23]. Mortality rates associated with IH following head trauma in patients using anticoagulants have been reported to range between 16% and 80% [22]. Various studies have documented the increased risk of hemorrhage associated with anticoagulant use after head trauma [22,24]. Ohm et al. reported that the use of antiplatelet agents nearly tripled the risk of trauma-related IH and mortality [22]. In the present study, IH was more common in patients using any medication and those using anticoagulants. The higher prevalence of IH in patients with comorbid conditions, medication use, and anticoagulant use observed in the present study aligns with previous research findings.

In the present study, when antithrombotic drugs were individually analyzed, IH was found to be more common among patients using NOACs, while no significant difference was observed between those using other drugs and those not using any drugs. It is well-established in the literature that antiplatelet and anticoagulant drugs increase the risk of spontaneous IH. The combined use of these drugs has also been reported to further elevate the risk of IH [8]. A meta-analysis conducted by Caldeira et al. concluded that while NOACs are safer compared to standard antithrombotic therapies, they increase the risk of IH compared to placebo [6]. On the other hand, Wojcik et al. reported that warfarin use did not influence mortality outcomes following head trauma [25]. In the present study, an association was identified between NOAC use and IH. Although no relationship was found between other antithrombotic drugs and IH, further studies are required due to the limited number of cases in the present study.

Trauma is the most common cause of IH [26]. Falls and motor vehicle accidents are the most frequent mechanisms of trauma leading to traumatic brain injury [21,27]. Motor vehicle accidents account for most deaths [27]. In the study by Kasmaei et al., falls and motorcycle accidents were reported as risk factors for mortality in patients with traumatic brain injury [16]. However, Sadegh et al. found no association between the cause of injury and positive CT findings [21]. In the present study, when cases were analyzed based on trauma mechanisms, IH was more common in those involved in traffic accidents, while no significant association was found between falls and IH. The higher energy of trauma in traffic accidents compared to falls may explain why traffic accidents were associated with IH in the present study.

In patients with head trauma, primary injury refers to direct mechanical damage, such as fractures and hemorrhage caused by trauma. Secondary injury involves blood–brain barrier disruption, production of reactive oxygen species, oxidative stress, metabolic dysfunction, and inflammation, which worsens the prognosis of patients [28]. Vital signs, laboratory parameters, and monitoring values are used to assess secondary injuries [10]. In the literature, hypertension, hypotension, hypoxia, and hypercapnia have been associated with increased mortality in patients with head trauma [27,29,30]. Laboratory abnormalities are common in traumatic brain injury patients, with elevated blood glucose levels, prolonged prothrombin time, and low hemoglobin and platelet levels reported to be associated with poor prognosis [31]. In the present study, no significant differences were observed in vital signs (SBP, DBP, pulse, respiratory rate, and SPO_2_) with respect to the presence of hemorrhage on brain CT scans. This may be because the vital signs of the patients were within normal values. Additionally, no significant differences were found between laboratory values at ED admission (glucose, ethanol, INR, aPTT, Hb, and platelet count) and the presence of hemorrhage on admission brain CT scans. However, cases with aPTT > 34 s showed a higher incidence of increased/new hemorrhage on follow-up brain CT scans. This finding may be explained by aPTT reflecting coagulation cascade abnormalities, which increase the risk of hemorrhage.

GCS is the most commonly used scoring system for determining the severity, prognosis, and follow-up of patients with head trauma in emergency settings [32]. In cases of head trauma and IH, a lower GCS is associated with a worse prognosis, as well as increased morbidity and mortality risk [9,33]. While patients with mild head trauma typically have higher GCS scores and intact consciousness, moderate to severe head trauma cases are marked by lower GCS scores, impaired consciousness, and the presence of neurological deficits [10,34,35]. Consistent with these findings, the present study also found that hemorrhage was more prevalent in non-minor head trauma cases. In a study conducted by Gómez et al., patients with a GCS score of 13–14 or those with altered consciousness at initial presentation had a 1.2 times greater likelihood of abnormal CT findings, hospitalization, and delayed hemorrhage compared to those with a GCS score of 15 [36]. Similarly, Kasmaei et al. reported that a GCS score < 9 was a risk factor for mortality in head trauma patients [16]. In the present study, IH was more common among patients presenting with altered consciousness at ED admission or pathological findings in central nervous system examinations. Additionally, patients with changes observed during physical examinations at the 6th hour, 24th hour, and 30th day were more likely to have IH on brain CT at admission. Sadegh et al. also reported that loss of consciousness and confusion were associated with abnormal findings on brain CT [20]. In cases of central nervous system damage and altered consciousness, GCS scores are typically lower. It is expected that patients with low GCS scores would have worse prognoses and show changes during follow-up physical examinations. The results obtained in the present study support these findings.

IH is a life-threatening condition that can lead to serious complications such as neurological deficits and infarction [4,9]. A study from Iran investigating head trauma patients in emergency settings reported that 23.3% of the patients died, and IH was identified as a risk factor for mortality in those with traumatic brain injuries [16]. In the present study, the prognosis of head trauma patients with IH was expected to be worse than that of patients without hemorrhage. As expected, hospitalization and mortality were more frequently observed in cases where hemorrhage was detected on initial brain CT.

Delayed IH is a potential complication in head trauma patients using anticoagulants [7]. Imaging with follow-up brain CT is recommended for patients using antiplatelet or anticoagulant drugs, as these medications increase the risk of delayed hemorrhage after head trauma [37,38]. Wong et al. reported that patients using clopidogrel had a higher likelihood of delayed hemorrhage compared to those not using it [39]. Zeeshan et al. noted that patients using NOACs experienced delayed hemorrhages more frequently than those using warfarin [40]. Beynon et al. found that while the rate of IH in patients using rivaroxaban after head trauma was similar to those not using antithrombotics, delayed hemorrhage was more common in the rivaroxaban group [41]. In a meta-analysis by Chauny et al., the incidence of IH on follow-up CT performed 24 h later was estimated to be 0.60% in patients with mild traumatic brain injury and normal initial CT findings who were on warfarin [7]. In the present study, no significant difference in antithrombotic use was observed between cases with newly detected hemorrhage and/or increased hemorrhage on follow-up CT and those without. This may be attributed to the limited number of cases in the study.

## 5. Limitations

There are certain limitations in this study. First, the number of cases with IH and mortality following head trauma was small. Therefore, statistical analyses of the factors affecting mortality and outcomes could not be performed. Second, larger studies with more cases are needed to evaluate the factors influencing IH, mortality, and outcomes in head trauma patients.

## 6. Conclusions

In the present study, advanced age, presence of chronic diseases, use of any medication, use of anticoagulants, use of NOACs, presence of pathological findings on physical examination, and non-minor head trauma were found to be associated with IH in head trauma patients. The only factor that showed a significant relationship with increased or newly developed hemorrhage on follow-up brain CT was prolonged aPTT.

Emergency departments frequently see a high volume of patients presenting with head trauma. It is crucial to have a good understanding of the management of head trauma patients and patients using antithrombotic agents to avoid overlooking risky situations and unnecessary tests and follow-ups. More comprehensive studies are needed to ensure standardization in the management of head trauma patients using antithrombotic agents.

## 7. Transparency, Rigor and Reproducibility Summary

This was a prospective observational study involving adult patients presenting to the emergency department (ED) with head trauma. The study design and analytic plan were not formally pre-registered in an external repository due to the observational nature of the study and absence of such requirements at the time of ethical approval. The analysis plan was developed prior to data collection and adhered to throughout the study.

A planned sample size was not determined by a priori power analysis; instead, all eligible patients presenting within a one-year period were included. A total of 1453 patients presented with head trauma; 895 were excluded due to being <18 years old and 2 due to pregnancy. Ultimately, 556 patients were included in the analysis. Of these, 398 underwent brain CT, and 41 of those received follow-up CT imaging. Data from all patients who met the inclusion criteria were analyzed without imputation. No data were excluded due to poor quality or technical error.

Blinding was not applicable for patient inclusion or initial clinical assessment due to the real-world clinical nature of the study. However, statistical analysis was conducted by investigators who were blinded to patient identifiers and treatment outcomes. Data collection was performed using the hospital information system and standardized emergency department protocols.

Clinical outcomes such as Glasgow Coma Scale (GCS) and Glasgow Outcome Scale scores were recorded by trained emergency physicians using validated scales that are established standards in traumatic brain injury research. Laboratory and imaging assessments were conducted as part of routine clinical care using institutional protocols. Data acquisition took place between 27 February 2019 and 27 February 2020. Data were collected from both the ED records and follow-up visits or phone interviews. Vital signs and laboratory data were collected at the time of ED presentation, and follow-up data were collected at 6 h, 24 h, and 30 days post-admission.

Assumptions for statistical analyses, including independence and normality of distribution, were assessed. Categorical variables were analyzed using the Chi-square test. Statistical review was performed by the lead author in collaboration with a statistician using IBM SPSS Statistics Version 21. Effect sizes and confidence intervals were reported where appropriate. Due to the exploratory nature of the study, corrections for multiple comparisons were not applied.

This study did not include replication or external validation. The study team plans to pursue additional multicenter validation in future prospective research. De-identified individual-level data from this study will be made available by request to the corresponding author, subject to institutional review board approval. Analytic code used for the statistical analysis is available upon request. The manuscript will be made publicly available upon publication under the Creative Commons CC-BY 4.0 license.

## Figures and Tables

**Table 1:** Relationship Between Demographic and Emergency Admission Characteristics of Cases and the Presence of Hemorrhage on Brain Computed Tomography (CT).

Variables		*n* (%)	Hemorrhage on Brain CT Presence *n* (%)	*p* *
Gender	Female	169 (42.5)	8 (4.7)	0.862
	Male	229 (57.5)	10 (4.4)	
Age (years)	≤59	244 (61.3)	7 (2.9)	0.046
	≥60	154 (38.7)	11 (7.1)	
Presence of Comorbidities		177 (44.5)	14 (7.9)	0.004
Use of Any Medication		167 (42.0)	14 (8.4)	0.002
Use of Antithrombotic Drugs		105 (26.4)	8 (7.6)	0.098
Use of Antiplatelet Drugs		78 (19.6)	5 (6.4)	0.366
Use of Anticoagulant Drugs		29 (7.3)	4 (13.8)	0.034
Use of Warfarin		19 (4.8)	2 (10.5)	0.210
Use of novel oral anticoagulant		8 (2.0)	2 (25.0)	0.046
Use of Antiplatelet + Anticoagulant Drugs		2 (0.5)	1 (50)	0.089
Mechanism of Trauma	Traffic accident	60 (15.1)	6 (10.0)	0.039
	Fall	223 (56.0)	12 (5.4)	0.352
Head trauma	Minor	391 (98.2)	15 (3.8)	0.002
	Non-minor	7 (1.7)	3 (42.8)	

* Statistical analysis using Chi-square test.

**Table 2:** Relationship Between Physical Examination Findings at Emergency Department (ED) Admission and Changes in Follow-Up Examinations with the Presence of Hemorrhage on Brain Computed Tomography (CT).

Physical Examination Findings	*n* (%)	Hemorrhage in Brain CT Presence *n* (%)	*p* *
Altered Consciousness at ED Admission	13 (3.3)	4 (30.8)	0.002
Pathological Findings on Central Nervous System Examination at ED Admission	13 (3.3)	5 (38.5)	<0.001
Changes in Findings at the 6th-hour Examination	4 (2.1)	2 (50.0)	0.030
Changes in Findings at the 24th-hour Examination	6 (5.9)	3 (50.0)	0.034
Changes in Findings at the 30th-Day Examination	16 (4.0)	5 (31.2)	<0.001

* Statistical analysis using Chi-square test.

**Table 3:** Distribution of Clinical Outcomes Based on the Presence of Hemorrhage on Brain Computed Tomography (CT) at Admission.

Clinical Outcome	*n* (%)	Hemorrhage in Brain CT Presence *n* (%)	*p* *
Discharge/Follow-up refusal	350 (87.9)	2 (0.6)	<0.001
Hospitalization/Intensive Care Unit Admission/Death	48 (12.0)	16 (33.3)	

* Statistical analysis using Chi-square test.

**Table 4:** Relationship Between Laboratory Values at Emergency Department Admission and Increased/New Hemorrhage on Follow-Up Brain Computed Tomography (CT).

Laboratory Values *		*n* (%)	Increased/New Hemorrhage on Follow-Up Brain CT *n* (%)	*p* **
Glucose	>180	2 (5.3)	1 (50.0)	0.339
	≤180	36 (94.7)	6 (16.7)	
INR	>1.4	9 (30.0)	2 (22.2)	−1.000
	≤1.4	21 (70.0)	4 (19.0)	
aPTT	>34	9 (30.0)	4 (44.4)	0.046
	≤34	21 (70.0)	2 (9.5)	

* Ethanol, hemoglobin, and platelet values could not be included in the analysis due to the small number of cases. ** Statistical analysis using Chi-square test.

## Data Availability

The data supporting the findings of this study are available within the article and can be provided by the corresponding author upon reasonable request.

## References

[1] Frieden T.R., Ikeda R., Hunt R.C. Traumatic Brain Injury in the United States Emergency Department Visits, Hospitalizations and Deaths 2002–2006. https://www.cdc.gov/TraumaticBrainInjury.

[2] Ja L., Rutland-Brown W., Wald M.M. (2006). The epidemiology and impact of traumatic brain injury: A brief overview. J. Head Trauma Rehabil..

[3] Head Trauma: Background, Pathophysiology, Etiology. 14 June 2021. https://emedicine.medscape.com/article/433855-overview.

[4] Abdelmalik P.A., Draghic N., Ling G.S.F. (2019). Management of moderate and severe traumatic brain injury. Transfusion.

[5] Feigin V.L., Theadom A., Barker-Collo S., Starkey N.J., McPherson K., Kahan M., Dowell A., Brown P., Parag V., Kydd R. (2013). Incidence of traumatic brain injury in New Zealand: A population-based study. Lancet Neurol..

[6] Caldeira D., Barra M., Pinto F.J., Ferreira J.J., Costa J. (2015). Intracranial hemorrhage risk with the new oral anticoagulants: A systematic review and meta-analysis. J. Neurol..

[7] Chauny J.-M., Marquis M., Bernard F., Williamson D., Albert M., Laroche M., Daoust R. (2016). Risk of Delayed Intracranial Hemorrhage in Anticoagulated Patients with Mild Traumatic Brain Injury: Systematic Review and Meta-Analysis. J. Emerg. Med..

[8] Naidech A.M. (2011). Intracranial hemorrhage. Am. J. Respir. Crit. Care Med..

[9] Tenny S., Thorell W. (2022). Intracranial Hemorrhage. StatPearls.

[10] ATLS Advanced Trauma Life Support 10th Edition 2018—ATLS ADVANCED TRAUMA—Docsity. https://www.docsity.com/pt/atls-advanced-trauma-life-support-10th-edition-2018/4905996/.

[11] Aydoğdu S., Güler K., Bayram F., Altun B., Derici Ü., Abacı A., Tükek T., Sabuncu T., Arıcı M., Erdem Y. (2019). 2019 Turkish Hypertension Consensus Report. Turk. Kardiyol. Dern. Ars..

[12] Akansel N., Yildiz H. (2010). Pulse Oksimetre Değerlerinin Güvenilir Olması İçin Neleri Bilmeliyiz?. Turk. Klin. J. Anest. Reanim..

[13] Balestreri M., Czosnyka M., Chatfield D.A., Steiner L.A., Schmidt E.A., Smielewski P., Matta B., Pickard J.D. (2004). Predictive value of Glasgow Coma Scale after brain trauma: Change in trend over the past ten years. J. Neurol. Neurosurg. Psychiatry.

[14] Caceres J.A., Goldstein J.N. (2012). Intracranial hemorrhage. Emerg. Med. Clin. N. Am..

[15] Leitgeb J., Mauritz W., Brazinova A., Janciak I., Majdan M., Wilbacher I., Rusnak M. (2011). Effects of gender on outcomes after traumatic brain injury. J. Trauma Acute Care Surg..

[16] Monsef Kasmaei V., Asadi P., Zohrevandi B., Raouf M.T. (2015). An Epidemiologic Study of Traumatic Brain Injuries in Emergency Department. Emergency.

[17] Testa J.A., Malec J.F., Moessner A.M., Brown A.W. (2005). Outcome after traumatic brain injury: Effects of aging on recovery. Arch. Phys. Med. Rehabil..

[18] Edwards P., Arango M., Balica L., Cottingham R., El-Sayed H., Farrell B., Fernandes J., Gogichaisvili T., Golden N., Hartzenberg B. (2005). Final results of MRC CRASH, a randomised placebo-controlled trial of intravenous corticosteroid in adults with head injury-outcomes at 6 months. Lancet.

[19] Hashmi A., Ibrahim-Zada I., Rhee P., Aziz H., Fain M.J., Friese R.S., Joseph B. (2014). Predictors of mortality in geriatric trauma patients: A systematic review and meta-analysis. J. Trauma Acute Care Surg..

[20] Mosenthal A., Lavery R., Addis M., Kaul S., Ross S., Marburger R., Deitch E., Livingston D. (2002). Isolated Traumatic Brain Injury: Age Is an Independent Predictor of Mortality and Early Outcome. J. Trauma Inj. Infect. Crit. Care.

[21] Sadegh R., Karimialavijeh E., Shirani F., Payandemehr P., Bahramimotlagh H., Ramezani M. (2016). Head CT scan in Iranian minor head injury patients: Evaluating current decision rules. Emerg. Radiol..

[22] Ohm C., Mina A., Howells G., Bair H., Bendick P. (2005). Effects of antiplatelet agents on outcomes for elderly patients with traumatic intracranial hemorrhage. J. Trauma Acute Care Surg..

[23] Narum S., Brørs O., Stokland O., Kringen M.K. (2016). Mortality among head trauma patients taking preinjury antithrombotic agents: A retrospective cohort analysis from a Level 1 trauma centre. BMC Emerg. Med..

[24] Batchelor J.S., Grayson A. (2012). A meta-analysis to determine the effect of anticoagulation on mortality in patients with blunt head trauma. Br. J. Neurosurg..

[25] Wojcik R., Cipolle M.D., Seislove E., Wasser T.E., Pasquale M.D. (2001). Preinjury warfarin does not impact outcome in trauma patients. J. Trauma Acute Care Surg..

[26] Heit J.J., Iv M., Wintermark M. (2017). Imaging of Intracranial Hemorrhage. J. Stroke.

[27] Vella M.A., Crandall M.L., Patel M.B. (2017). Acute Management of Traumatic Brain Injury. Surg. Clin. N. Am..

[28] Mutch C.A., Talbott J.F., Gean A. (2016). Imaging Evaluation of Acute Traumatic Brain Injury. Neurosurg. Clin. N. Am..

[29] Jain V., Choudhary J., Pandit R. (2019). Blood Pressure Target in Acute Brain Injury. Indian J. Crit. Care Med. Peer-Rev. Off. Publ. Indian Soc. Crit. Care Med..

[30] Butcher I., Maas A.I.R., Lu J., Marmarou A., Murray G.D., Mushkudiani N.A., McHugh G.S., Steyerberg E.W. (2007). Prognostic value of admission blood pressure in traumatic brain injury: Results from the IMPACT study. J. Neurotrauma.

[31] Van Beek J.G.M., Mushkudiani N.A., Steyerberg E.W., Butcher I., McHugh G.S., Lu J., Marmarou A., Murray G.D., Maas A.I.R. (2007). Prognostic value of admission laboratory parameters in traumatic brain injury: Results from the IMPACT study. J. Neurotrauma.

[32] Keser N., Döşoğlu M.S. (2019). Determination of Prognostic Factors in Cerebral Contusions. Bagcilar Med. Bull..

[33] Collaborators M.C.T. (2008). Predicting outcome after traumatic brain injury: Practical prognostic models based on large cohort of international patients. BMJ.

[34] Heegaard W., Biros M. (2007). Traumatic brain injury. Emerg. Med. Clin. N. Am..

[35] Servadei F., Teasdale G., Merry G., Neurotraumatology Committee of the World Federation of Neurosurgical Societies (2001). Defining acute mild head injury in adults: A proposal based on prognostic factors, diagnosis, and management. J. Neurotrauma.

[36] Gómez P.A., Lobato R.D., Ortega J.M., De La Cruz J. (1996). Mild head injury: Differences in prognosis among patients with a Glasgow Coma Scale score of 13 to 15 and analysis of factors associated with abnormal CT findings. Br. J. Neurosurg..

[37] Joseph B., Sadoun M., Aziz H., Tang A., Wynne J.L., Pandit V., Kulvatunyou N., O’Keeffe T., Friese R.S., Rhee P. (2014). Repeat head computed tomography in anticoagulated traumatic brain injury patients: Still warranted. Am. Surg..

[38] Brown C.V.R., Zada G., Salim A., Inaba K., Kasotakis G., Hadjizacharia P., Demetriades D., Rhee P. (2007). Indications for routine repeat head computed tomography (CT) stratified by severity of traumatic brain injury. J. Trauma Acute Care Surg..

[39] Wong D.K., Lurie F., Wong L.L. (2008). The effects of clopidogrel on elderly traumatic brain injured patients. J. Trauma Acute Care Surg..

[40] Zeeshan M., Jehan F., O’Keeffe T., Khan M., Zakaria E.R., Hamidi M., Gries L., Kulvatunyou N., Joseph B. (2018). The novel oral anticoagulants (NOACs) have worse outcomes compared with warfarin in patients with intracranial hemorrhage after TBI. J. Trauma Acute Care Surg..

[41] Beynon C., Potzy A., Sakowitz O.W., Unterberg A.W. (2015). Rivaroxaban and intracranial haemorrhage after mild traumatic brain injury: A dangerous combination?. Clin. Neurol. Neurosurg..

